# SLIT2/ROBO1-miR-218-1-RET/PLAG1: a new disease pathway involved in Hirschsprung's disease

**DOI:** 10.1111/jcmm.12454

**Published:** 2015-03-19

**Authors:** Weibing Tang, Junwei Tang, Jun He, Zhigang Zhou, Yufeng Qin, Jingjing Qin, Bo Li, Xiaoqun Xu, Qiming Geng, Weiwei Jiang, Wei Wu, Xinru Wang, Yankai Xia

**Affiliations:** a State Key Laboratory of Reproductive Medicine, Institute of Toxicology, School of Public Health, Nanjing Medical UniversityNanjing, China; b Laboratory of Modern Toxicology (Nanjing Medical University), Ministry of EducationChina; c Department of Pathology, Thomas Jefferson UniversityPhiladelphia, PA, USA; d Department of Pediatric Surgery, Nanjing Children's Hospital Affiliated Nanjing Medical UniversityNanjing, China

**Keywords:** intestine, gene regulation, neural development, genetic disorder

## Abstract

Hirschsprung's disease (HSCR) is a rare congenital disease caused by impaired proliferation and migration of neural crest cells. We investigated changes in expression of microRNAs (miRNAs) and the genes they regulate in tissues of patients with HSCR. Quantitative real-time PCR and immunoblot analyses were used to measure levels of miRNA, mRNAs, and proteins in colon tissues from 69 patients with HSCR and 49 individuals without HSCR (controls). Direct interactions between miRNAs and specific mRNAs were indentified *in vitro*, while the function role of miR-218-1 was investigated by using miR-218 transgenic mice. An increased level of miR-218-1 correlated with increased levels of SLIT2 and decreased levels of *RET* and *PLAG1*mRNA and protein. The reductions in *RET* and *PLAG1* by miR-218-1 reduced proliferation and migration of SH-SY5Y cells. Overexpression of the secreted form of *SLIT2* inhibited cell migration *via* binding to its receptor *ROBO1*. Bowel tissues from miR-218-1 transgenic mice had nerve fibre hyperplasia and reduced numbers of gangliocytes, compared with wild-type mice. Altered *miR-218-1* regulation of *SLIT2, RET and PLAG1* might be involved in the pathogenesis of HSCR.

## Introduction

Hirschsprung's disease (HSCR) is a gastrointestinal disorder with an incidence of 1:200–1:5000 live births, with males being four times more affected than females in short segment HSCR 1. It is characterized that the enteric neural crest cells (ENCCs) stop migrating and fail to reach the hindgut during embryogenesis from 5 to 12 weeks, which leads to the absence of ganglia cells in the intramural and submucosal along variable lengths of gastrointestinal tract 2. The aetiological studies of HSCR have shown that the development of disease is a complicated process involving both genetic factors and environmental conditions 3. In particular, genes controlling ENCC migration play important roles in the pathogenesis of HSCR 4. It is known that the ENCCs in the distal rectum migrate further than any other cells during embryogenesis. Thus, any aberration of survival, proliferation, migration or differentiation of ENCCs will result in aganglionosis of the distal gut 5. To date, more than 10 genes have been identified to be associated with the pathogenesis of HSCR 6. *RET* proto-oncogene known as tyrosine kinase receptor, is widely expressed in neural crest cell, endocrine system and urinogenital system 6. *RET* protein is crucial for the development of enteric neuron cells. Some evidence have shown that mutations that lead to a reduction of RET expression could result in HSCR 7. Furthermore, it has been reported that *RET* is associated with neural cell migration 8.

MiRNAs are small, non-coding RNA molecules of 19–25 nucleotides which have been reported to play important roles by regulating cell differentiation, proliferation, migration and apoptosis 9. miRNAs negatively regulate their target genes expression at the post-transcription level through binding to 3′ untranslated regions (UTRs) of their targets message RNAs 10. To date, more than 800 miRNAs have been identified in mammalian cells 11. Many of them have also been implicated in cancer development and metastasis 12. In addition, certain miRNAs have been found in the central neural system during embryonic development 13. However, to our knowledge, the role of miRNAs in HSCR disease is not known yet.

miRNAs are transcribed in parallel with their host transcripts, and the two different transcription classes of miRNAs (‘exonic’ and ‘intronic’) identified have been reported to play important roles in the pathogenesis of different diseases 14. For example, the expression level of miR-126 was directly controlled by its host gene *EGFL7* through epigenetic changes 15. miR-103 and miR-107, hosted by pantothenate kinase genes, are proposed to regulate cellular lipid metabolism 16. So far, few miRNAs and their related host genes are observed to be involved in embryonic peripheral nervous system development, especially in the enteric nervous system (ENS) development.

Slit homologue 2/Roundabout homologue 1(SLIT2/ROBO1) pathway is closely related with cell migration 17. Along with an evolutionary conserved role in axon guidance, SLIT2/ROBO1 pathway has a key function in nervous system, especially in neural crest cell migration 18. The full length of the secreted protein SLIT2 can be cleaved into two smaller fragments, a 140 kD N-terminal product (N-SLIT2) and a 50–60 kD C-terminal product (C-SLIT2). N-SLIT2 is the fragment responsible to bind ROBO1, a single-pass transmembrane receptor of SLIT2 19. In this study, we began our study based on the potential miRNA regulating RET in HSCR by bioinformatics prediction. The results indicated that miR-218-1 was the top one miRNA, thus, we investigated the roles of miR-218-1, SLIT/ROBO1 in HSCR disease development by using human tissues, cells and a transgenic mice model.

## Materials and methods

### Ethics statement and samples collection

This study was approved by the Institutional Ethics Committee of Nanjing Medical University, and it was performed under compliance with the government policies and the Helsinki Declaration. Both HSCR and control group samples were collected after informed consent was obtained from their guardians. A total of 69 HSCR colon tissues were obtained from HSCR patients who had acquired surgical treatment in Nanjing Children's Hospital Affiliated to Nanjing Medical University from October 2009 to May 2012 (NJMU Birth Cohort). The 69 patients in our study consisted of 42 short segment patients and 27 long segment patients. All the patients were diagnosed by barium enema and anorectal manometry evaluation before surgical procedures. After surgery, pathological analysis was performed for definite diagnosis. Colon tissues of 49 controls were obtained from isolated patients that received surgical treatment because of intussusception or incarcerated and strangulated inguinal hernia without the ischaemia or necrosis. These patients did not have HSCR or other congenital malformation. All tissues collected were immediately frozen and stored at −80°C after surgery.

### Quantitative RT-PCR

Quantitative real-time polymerase chain reaction (qRT-PCR) was performed to determine the expression levels of miR-218-1 and mRNAs of all related genes. Total RNA was obtained from tissues using TRIzol reagent as described by the manufacturer (Invitrogen Life Technologies, Carlsbad, CA, USA). For mRNA detection, total RNAs (500 ng) were reverse transcribed using the reverse transcription kit (Takara, Tokyo, Japan). β-*actin* was used as an internal control. TaqMan® MicroRNA Assays ( Applied Biosystems, Foster City, CA, USA) was used as the probe for *has*-miR-218-1 and has-U6 which act as a normalized control. All the primer sequences are shown in Table S1. qRT-PCR was performed with ABI Prism 7900HT (Applied Biosystems) according to the manufacturer's instructions.

### Bioinformatics prediction

The method we used to predict the related potentially target RET was according to the prediction of the bioinformatical software online including Target Scan (www.targetscan.org), PicTar (pictar.mdc-berlin.de/) and miRNA.org (http://www.microrna.org/).

### Protein analysis

For Western blotting, total proteins were extracted from tissues or cultured cells using RIPA buffer containing protease inhibitors cOmplete, ULTRA, Mini, EDTA-free, EASYpack (Roche, Basel, Switzerland), while the membrane proteins were extracted from tissues by Mem-PER Eukaryotic Membrane Protein Extraction Reagent Kit (Thermo Scientific, Rockford, IL, USA). Equal amount of proteins (100 μg) were separated with 7.5%/12.5% SDS-PAGE and transferred to polyvinylidene fluoride membrane. Primary polyclonal antibodies including RET antibody (SC167), PLAG1 antibody (SC20320), SLIT2 antibody (SC26601) and ROBO1 antibody (SC16612) were purchased from Santa Cruz Biotechnology (Santa Cruz, CA, USA). The secondary antibodies were anti-rabbit or anti-goat HRP-linked were purchased from Santa Cruz Biotechnology. The blots were developed using ECL reagent (Millpore, Billerica, MA, USA). Equal amount of protein loading in each lane was confirmed using GAPDH antibody. The integrated density of the band was quantified by Image J software (National Institute of Mental Health, Bethesda, Maryland, USA).

### Cell culture and reagents

Human SH-SY5Y cell were obtained from American Type Culture Collection (ATCC, Manassas, VA, USA), and were cultured in DMEM (HyClone, Logan, UT, USA), supplemented with 10% foetal bovine serum, 100 U/ml penicillin, and 100 μg/ml streptomycin at 37°C, 5% CO_2_. Synthetic miRNA precursor molecules of miR-218-1, siRNA of *ROBO1* and negative control (GenePharma, Shanghai, China) were used in transfection experiments. Cells were treated with or without recombinant SLIT2-N protein (Peprotech, Rocky Hill, NJ, USA) at final concentrations of 100 ng/ml.

### Cell proliferation assays

Cell proliferation was assayed using EdU (5-Ethynyl-2′-deoxyuridine; Roche). EDU (Ribobio, Guangzhou, China) assay was used to examine the cell proliferation. Fluorescence staining was determined using a confocal microscope (Olympus, Tokyo, Japan). Each assay was performed in triplicate and repeated three times independently.

### Cell cycle and apoptosis analysis

Cells were transfected with miR-218 mimics as well as negative controls or treated with SLIT2-N for 48 hrs. All experiments were analysed by BD Biosciences FACS Calibur Flow Cytometry (BD Bioscience Pharmingen Inc., San Diego, CA, USA). The tests were repeated for three times with triplicate per experiment.

### Cell transwell assays

For those cells treated with miRNA or siRNA, after transfection for 48 hrs, cells were seeded at 1 × 10^6^ cells/ml with serum-free medium. For the cells treated with SLIT2-N, after 48 hrs treatment, 100 μl cell suspension with serum-free medium was seeded to the upper chamber, cells were stained with crystal violet staining solution (Beyotime, Nantong, China) then counted and photographed under 40× magnification (five views per well). Migrated cells were counted by using Image-pro Plus 6.0 while cell numbers of normal control group were normalized to 1. The integrated intensity of migrated cells was measured by MetaMorph/MetaXpress as well. All experiments were performed in triplicate.

### Dual-luciferase reporter assay

The 3′ UTR sequence of *RET* and *PLAG1* predicted to interact with miR-218 or a mutated sequence with the predicted target sites were inserted into the KpnI and SacI sites of pGL3 promoter vector (Genscript, Nanjing, China). These constructs were named *pGL3-RET*, *pGL3-PLAG1* and *pGL3-RET-mut*. For reporter assay, cells were plated onto 24-well plates and transfected with 100 ng of *pGL3-RET*, *pGL3-PLAG1* or *pGL3-RET-mut*, *pGL3-PLAG1-mut* 50 nM miR-218-1 mimics and control, respectively, using Lipofectamine 2000 (Invitrogen Corp). A Renilla luciferase vector pRL-SV40 (5 ng) was also cotransfected to normalize the differences in transfection efficiency. Transfection was repeated three times in triplicate.

### Transgenic mice

A fragment of DNA containing the precursor sequence of mmu-miR-218-1 was amplified and subcloned into the ScaI and BamHI sites of the pUBC CSH4 mMir218-1 constructs carrying the UBC promoter and BGH poly(A) signal. Transgenic mice were generated by pronuclear injection of the transgene into the C57BL/6 strain. Genomic DNA isolated from the tail was analysed. The positive miR-218-1 transgenic mice were identified by the successful PCR amplification. The detailed primer was MIR218-EF3/BGH-R (MIR218-EF3: 5′-CGAGGCAGGTCTTACTTGTCT-3′ BGH-R: 5′-AGAAGGCACAGTCGAGG-3′) with a product of 480 bp under the PCR condition of 94°C for 5 min., then 35 cycles of 94°C for 30 sec., 55°C for 30 sec., and 72°C for 30 sec.; 72°C, 10 min. The number of mice in F1 was total 83. Fifteen of 83 presented the successful overexpression of miR-218-1. Only one presented the post-natal death. Few mice in our study presented embryonic or post-natal lethality. The foetal gut was examined at E18.5. Adult male C57BL/6 mice (22–25 g) were used in this study. Mice were kept under standard animal room conditions with food and water *ad libitum* before the experimental procedures. Our experiments were approved by the Animal Care and Use Committee of Nanjing medical university, and were performed in accordance with the guidelines that were established by the Chinese Association for Laboratory Animal Sciences.

### Histology and immunohistochemistry

Bowel specimens were fixed in 4% paraformaldehyde, embedded in paraffin, sectioned at 4 μm and mounted on silane-coated glass slides. Sections were stained with haematoxylin and eosin according to standard protocols. Sections were deparaffinized and followed by rehydration steps through a graded ethanol series and distilled water, and then were treated with 3% H_2_O_2_ in methanol for 30 min to block the endogenous peroxidase activity. The sections were rinsed in PBS twice, 5 min. each time and incubated with 10% normal goat serum for 30 min. to block non-specific antibody binding. After washing, the samples were incubated with primary anti-rabbit antibody PGP9.5 (1:200, sc-25800; Santa Cruz Biotechnology) and cathepsin D (1:200, sc-10725; Santa Cruz Biotechnology) at 4°C overnight, and then washed in PBS for three times and then incubated with secondary antibodies. After that, the sections were stained with DAB according to manufacturer's protocols and mounted and photographed using a digitalized microscope camera (Nikon, Tokyo, Japan).

### Statistical analysis

The method of 2^−▵Ct^ was used to analyse the results of RT-PCR in all the experiments performed in this study. Statistical analysis was performed with STATA 9.2, and presented with Graph PAD prism software. Experimental data of tissue samples are presented as box plot of the median and range of log-transformed relative expression level which was analysed by Wilcoxon rank-sum (Mann–Whitney) test. The top and bottom of the box represent the 75th and 25th percentile. The whiskers indicate the 10th and 90th points. While the results obtained from experiment *in vitro* assays are presented as mean ± SEM from three separate experiments in triplicates per experiment, and the data were analysed by double-sided Student's *t*-test. Pearson correlation analysis was used to analyse the relationship of expression level of tissues between case and control group. Results were considered statistically significant at *P* < 0.05.

## Results

### Clinical information analysis

A total of 69 human HSCR colon tissue specimens collected from HSCR patients diagnosed with HSCR disease by pathological detection were used for this study. A total of 49 normal colon tissue specimens were used as controls. The clinical information, including age, gender rate (Male/Female) and bodyweight, was obtained from both HSCR patients and normal controls. The age of HSCR and control groups were 3.6 ± 0.24 and 3.2 ± 0.31 months old, respectively; the bodyweight were 5.9 ± 0.32 and 5.2 ± 0.26 kg, both of which had no statistical difference. The gender rate (Male/Female) of HSCR and control was 56/13 and 40/9, respectively, which matched the common gender rate of this disease in human beings.

### *RET* and *PLAG1* are down-regulated, whereas *miR-218-1* is up-regulated in HSCR patients

**Figure 1:**
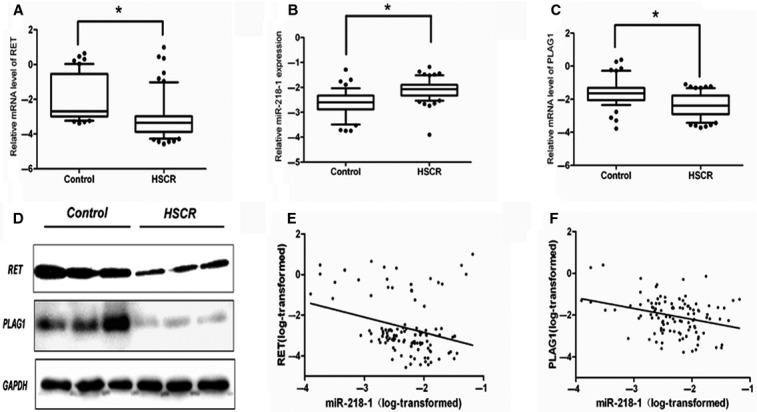
*RET* and *PLAG1* are up-regulated, whereas miR-218-1 is down-regulated in HSCR patients. (A) The mRNA levels of *RET* in human HSCR tissues (*n* = 69) and control tissues (*n* = 49) were evaluated by qRT-PCR. Data were presented as box plot of the median and range of log-transformed relative expression level. The top and bottom of the box represent the 75th and 25th percentile. The whiskers indicate the 10th and 90th points. * Significantly different compared with that of control (*P* < 0.05). (B) The expression levels of miR-218-1 in human HSCR tissues and control tissues. (C and D) The expression levels of RET and PLAG1 in human HSCR tissues (three representative samples from both groups are shown). (E and F) RET and PLAG1 are down-regulated, whereas miR-218-1 is up-regulated in HSCR patients, *P* = 0.003, *R* = −0.28, *P* = 0.001, *R* = −0.31, respectively. Data were analysed using Pearson correlation analysis with natural log-transformed expression levels.

As *RET* was one of the most important genes related to HSCR, we used RT-PCR and Western blotting to examine *RET* expression levels in HSCR samples and control samples. RET expression levels in HSCR samples were significantly lower than those in the controls, *P* = 5.53 × 10^−9^ (Fig.1A) which was consistent with their protein expression levels. We used bioinformatics methods to predict related miRNAs which potentially target *RET* gene. The results indicated that only miR-218-1 was predicted to target *RET* by all three databases, which prompted us to investigate the expression level of miR-218-1 in HSCR samples. Interestingly, miR-218-1 in HSCR samples were found to be significantly higher in HSCR than in controls samples (*P* = 8.02 × 10^−8^) as shown in Figure1B, indicating that *RET* and miR-218-1 might be involved in the pathological development of HSCR disease.

We also selected another five candidate target genes of miR-218-1 mRNA, which showed a high score in all of the bioinformatics software described above for expression analysis (Table S1). Among the five candidate genes predicted, only *PLAG1* exhibited a decreased mRNA level in HSCR samples compared with the control samples (*P* = 1.21 × 10^−6^, see Fig.1C). The other four genes, *SLC1A2*, *TMEM25*, *ZDHHC8* and *EIF5A2*, showed no statistical difference, as indicated by *P* = 0.62, 0.72, 0.75, 0.74, respectively (data not shown). Furthermore, the Western blotting assay confirmed the down-regulation of PLAG1 in HSCR samples at protein level (Fig.1D). Integrated density was presented in Figure S4A.

Next, we analysed the relationship between miR-218-1 and *RET* relative expression levels in patients' samples and control samples. An inverse correlation was observed between *RET* expression and miR-218-1 expression, *P* = 0.003, *R* = −0.28 (Fig.1E). These results suggested that the level of *RET* could be regulated by miR-218-1. Similarly, statistical analysis of the qRT-PCR results also revealed a significant inverse correlation between the levels of *PLAG1* mRNA and miR-218-1, *P* = 0.001, *R* = −0.31 (Fig.1F), suggesting miR-218-1 regulates both *RET* and *PLAG1* in HSCR.

### Overexpression of miR-218-1 reduced RET and PLAG1 proteins expression resulting in inhibition of cell proliferation and migration

**Figure 2:**
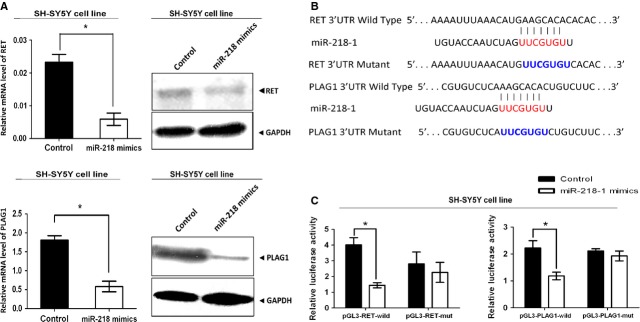
Overexpression of miR-218-1 reduced RET and PLAG1 proteins expression. (A) SH-SY5Y cells were transfected with 50 nM miR-218 mimics for 48 hrs. qRT-PCR was performed to evaluate the mRNA level of *RET* and *PLAG1* (left panel). RET and PLAG1 protein expression levels were analysed by Western blotting (right panel). (B) Sequence alignment of human miR-218-1 with 3′ UTR of *RET* or *PLAG1*. Bottom: mutations in the 3′ UTR of *RET* and *PLAG1* to create the mutant luciferase reporter construct. (C) Cells were cotransfected with miR-218-1 mimics or miR-control, renilla luciferase vector pRL-SV40 and *RET* or *PLAG1* 3′ UTR luciferase reporters for 48 hrs. Both firefly and Renilla luciferase activities are measured in the same sample. Firefly luciferase signals were normalized with Renilla luciferase signals. * indicates significant difference compared with that of control cells (*P* < 0.05). All tests were performed in triplicate and presented as mean ± SE.

To determine the effect of miR-218-1 on RET and PLAG1 expressions, we overexpressed miR-218-1 in SH-SY5Y cell line. The transfection efficiency was examined by fluorescence microscope (Fig. S1A). As expected, overexpression of miR-218-1 decreased RET and PLAG1 both at mRNA and protein levels (Fig.2A). Integrated density was presented in Figure S4B. To investigate whether the inhibitory effect was because of direct binding of miR-218-1 to the 3′ UTR regions of *RET* and *PLAG1*, we performed miRNA luciferase reporter assay by constructing the wild-type and mutant type luciferase reporter plasmids containing the binding region of the 3′ UTR of *RET* or *PLAG1* mRNAs. We found that cotransfection of miR-218-1 mimics and *pGL3-RET* or *pGL3-PLAG1* 3′ UTR reporter plasmids significantly decreased luciferase activity in cell lines when compared to the control (Fig.2B and C) suggesting that miR-128-1 directly targets both RET and PLAG1.

As PLAG1 has been reported to be associated with cell proliferation, we further conducted the experiment to investigate the potential role of PLAG1 in the pathogenesis of HSCR. As described above, the expression level of PLAG1 was reduced by siRNA as confirmed in Figure S3A. The EDU assay was used to detect the proliferation in SH-SY5Y cell line. The results showed that the proliferation ability of cells was suppressed because of the decreased level of PLAG1 (Figure S3B).

**Figure 3:**
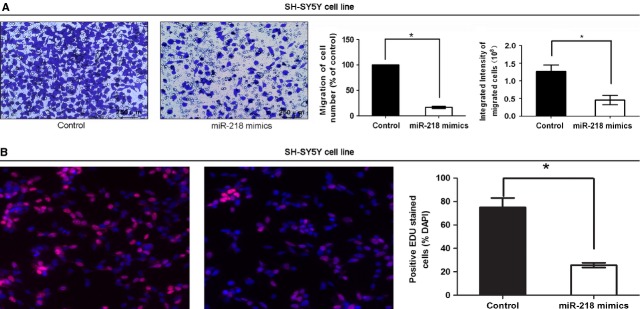
Cytobiology change after treating cells with *miR-218* mimics. (A) Transwell assay was performed as described in Materials and methods. The representative images of invasive cells at the bottom of the membrane stained with crystal violet were visualized as shown (left). The quantifications of cell migration were presented as percentage migrated cell numbers and the integrated intensity of migrated cells (right). * indicates significant difference compared with control group (*P* < 0.05). (B) EDU assay was performed as described. The integrated density was presented with mean ± SE. * indicates significant difference compared with control group *P* < 0.05.

To detect the functional roles of miR-218-1, we then examined the effect of miR-218-1 on cell proliferation and cell migration. A suppressive effect was observed in cell migration and proliferation in SH-SY5Y cell line by using the transwell assay and EDU assay respectively (Fig.3A and B). Both the number of migrated cells and the integrated intensity of migrated cells were significantly lower in miR-218-1 overexpressing cells, suggesting that cell migration was suppressed by miR-218-1.

### *SLIT2*, the host gene of miR-218-1, and *SLIT2/ROBO1* pathway in HSCR

**Figure 4:**
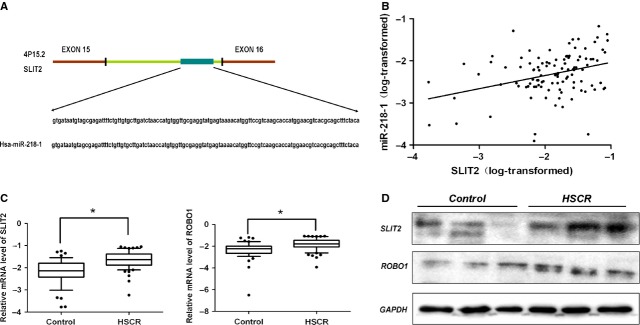
Up-regulated SLIT2/ROBO1 pathway played an important role in HSCR. (A) The genomic locations of *miR-218-1* gene hosted in the intron 15 (274664–274773) of *SLIT2*. (B) Pearson correlation analysis was performed to evaluated the relationship between expression levels of *SLIT2*mRNA and miR-218-1 (data with natural log transformed), *P* = 0.0003, *R* = 0.34. (C) The expression levels of *SLIT2* in human HSCR tissues and control tissues as described above. * Significantly different compared with that of control (*P* < 0.05). (D) The proteins levels of SLIT2, ROBO1 in HSCR tissues and control tissues were determined by Western blotting. Three representative samples from each group were shown. GAPDH was used as a loading control.

The miR-218-1 gene resides in the intron 15 (274664–274773) of *SLIT2* gene 20, which acts as the host gene of miR-218-1 (Fig.4A). The correlation analysis showed that a significant positive correlation was observed between the levels of miR-218-1 and *SLIT2*, *P* = 0.0003, *R* = 0.34 (Fig.4B), indicating miR-218-1 was transcribed together with its host genes *SLIT2*. We assessed the expression status of *SLIT2* and its receptor *ROBO1* in HSCR tissue samples. As shown in Figure4C, both *SLIT2* and its receptor *ROBO1* mRNAs displayed a higher level in HSCR, *P* = 5.72 × 10^−8^ and 4.47 × 10^−6^, respectively. Besides, a positive correlation was observed between *SLIT2* expression and *ROBO1* expression, *P* = 0.03, *R* = 0.21 (Fig.4C). The protein levels of SLIT2 and ROBO1 showed the same tendency as well (Fig.4D), suggesting *SLIT2*/*ROBO1* pathway might also play a vital role in the pathogenesis of HSCR. Integrated density was presented in Figure S4A.

### Overexpression of SLIT2 suppressed cell migration *via* SLIT2/ROBO1 pathway

The above results prompted us to hypothesize that SLIT2, the host gene of miR-218-1 may also be important in the pathogenesis of HSCR. To validate this hypothesis, we investigated the effects of SLIT2 on cell growth, migration, apoptosis and cell cycle as well. *SLIT2* can be cleaved into Slit2-C, a diffusible fragment, and Slit2-N, a protein tightly binding to the cell membrane to exert its function. We applied an exogenous SLIT2-N to determine the functional effect of *SLIT2 in vitro*.

EDU assays were performed to examine the effect of Slit2-N on cell growth. After being treated with 100 ng/ml of Slit2-N for 24 hrs, the SH-SY5Y cells showed no significant difference compared with the control (Fig. S2A). We also evaluated the effect of SLIT2-N on apoptosis and cell cycle. As shown in Figure S2B and C, Slit2-N treatment for 24 hrs had no significant effect on cell apoptosis and cell cycle.

**Figure 5:**
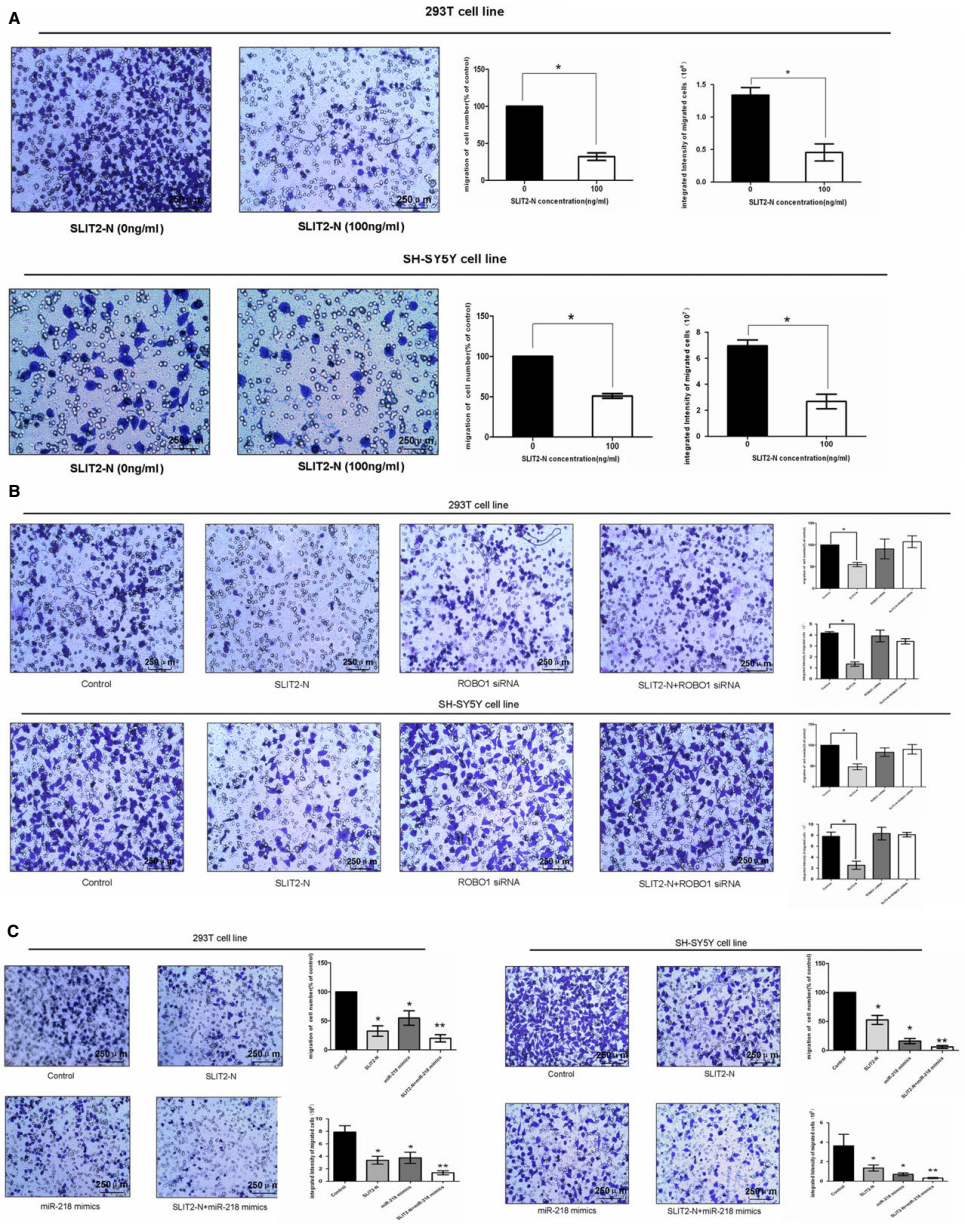
Overexpression of SLIT2 *via*SLIT2/ROBO1 pathway combining with miR-218-1 suppressed cell migration. (A) Transwell assay was performed as described in Materials and methods. The representative images of invasive cells at the bottom of the membrane stained with crystal violet were visualized as shown (left). The quantifications of cell migration were presented as percentage migrated cell numbers and the integrated intensity of migrated cells (right). (B) Cells were treated with 100 ng/ml SLIT2-N, *ROBO1* siRNA, SLIT2-N+ROBO1 siRNA and normal control for 48 hrs. The representative images of invasive cells at the bottom of the membrane stained with crystal violet were visualized as shown (left). The quantifications of cell migration were presented as the percentage of migrated cell numbers and the integrated intensity of migrated cells (right). (C) Cells were treated with 100 ng/ml SLIT2-N, miR-218 mimics, SLIT2-N+ miR-218 mimics and normal control for 48 hrs. The representative images of invasive cells at the bottom of the membrane stained with crystal violet were visualized as shown (left). The quantifications of cell migration were presented as percentage migrated cell numbers and the integrated intensity of migrated cells (right). All experiments were performed in triplicate and presented as mean ± SE. * indicates significant difference compared with control group (*P* < 0.05).

Boyden chamber migration assays were used to detect the migratory potential of both cells treated with recombinant SLIT2-N. As shown in Figure5A, the average number of migrated cells in SLIT2-N-treated cells was significantly lower than that of the migrated control cells (*P* < 0.01). Moreover, the integrated intensity of migrated cells measured by MetaMorph/MetaXpress was higher than control cells, which was consistent with the result of cell number counting.

To analyse whether the inhibitory effect on migration by SLIT2-N was mediated through SLIT2-ROBO1 interaction, RNAi experiments were performed. Three different siRNA oligonucleotides specifically against *ROBO1* were designed for experiments. The mRNA expression level of *ROBO1* was measured 48 hours after transfected with *ROBO1* siRNAs (Fig. S1B). After silencing *ROBO1* expression, application of 100 ng/ml SLIT2-N in the cells no longer affected cell migration. Similarly, *ROBO1* knockdown did not affect cell migration without SLIT2-N treatment (Fig.5B), demonstrating that effects of *SLIT2* are mediated by the ROBO1 receptor. As both miR-218-1 and *SLIT2* were demonstrated as migration suppressors in SH-SY5Y cells, we further aimed to determine whether the migration inhibition was caused only by miR-218-1 or SLIT2-N or both. Cells were treated with miR-218-1 mimics or SLIT2-N or both. The results showed either miR-218-1 mimics or SLIT2-N could suppress the migration ability of cells. Moreover, the combination of both enhanced the migration suppression compared with miR-218-1 mimics or SLIT2-N alone (Fig.5C), indicating miR-218-1 together with SLIT2 play the pivotal roles in the progress of HSCR.

### Overexpressed miR-218-1 down-regulated *Ret* and *Plag1* resulting in the absence of gangliocytes as well as the compensatory hyperplasia of the nerve fibres *in vivo*

**Figure 6:**
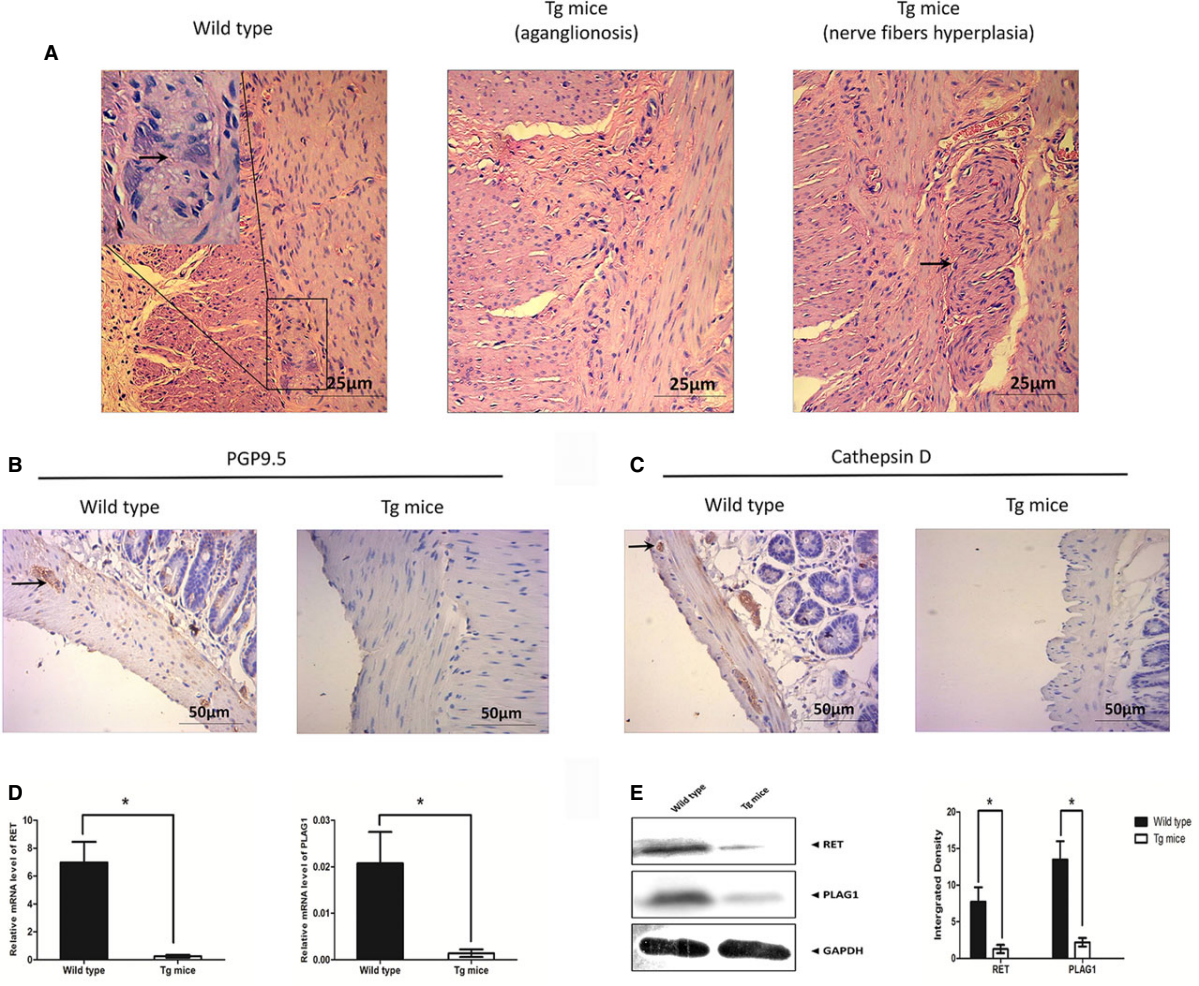
Effects of miR-218-1 transgenic mice *in vivo*. (A) Haematoxylin and eosin staining was used to detect the phenotype alternation in the rectum of miR-218-1 transgenic mice. Arrow in the left panel indicated the gangliocytes, the middle indicated the aganglionosis, while the right indicated the compensatory proliferation of the nerve fibres. (B and C) Immunohistochemistry assays were conducted to detect the expression of the biomarker of gangliocytes (PGP9.5 and cathepsin D). Arrow indicated positive staining of the proteins expressed in gangliocytes. (D and E) The mRNA and protein expression level of Ret and Plag1 were examined in miR-218-1 transgenic mice and wild-type mice. All experiments were performed in triplicate and presented as mean ± SE. * indicates significant difference compared with control group (*P* < 0.05). * indicates remarkable difference (*P* < 0.01).

The transgenic mice were generated as presented in Figure S4. Few mice in our study presented embryonic or post-natal lethality. The foetal gut was examined at E18.5. The haematoxylin and eosin-stained sections of the hindgut in positive mice showed a remarkable decrease in the gangliocytes which is an indicative of roles of miR-218-1 in pathogenesis of HSCR. As the consequence of the loss of ganglion cells, the nerve fibres were compensatory proliferated (Fig.6A). We also detected the ganglion cell of the transgenic mice afterbirth. The defect we detected after birth kept consist with the results of the mice during embryogenesis. Unfortunately, the defect was not clearly visible as abdominal distension or narrowed colon of mice, however, the ganglion cells was decreased in the miR-218-1 transgenic mice compared with the wild-type according to the results of the detailed stain of the colon.

To further confirm the loss of gangliocytes, we performed the immunohistochemistry assays to detect the expression of the biomarker of gangliocytes (PGP9.5 and cathepsin D). Representative images were shown in Figure6B and C. The expression of both PGP9.5 and cathepsin D were remarkably reduced compared with the wild-type (Fig. S4C).

Furthermore, we detected the expression levels of *Ret* and *Plag1* in the positive mice. As indicated in Figure 6D and E., both Ret and Plag1 were down-regulated in the miR-218-1 transgenic mice. Collectively, overexpression of miR-218-1 leads to the loss of ganglion cells mediated by Ret and Plag1 *in vivo*.

## Discussion

Hirschsprung's disease is a common malformation condition of the digestive tract in newborns. Aganglionosis attributes to the disorder of the ENS whereby ganglion cells fail to innervate a variable length of the gastrointestinal tract. The ENCCs which have a leading role in the ENS colonization, are very important in the development of HSCR. Derived from vagus nerve, the ENCCs move along the way of the vagus nerves, enter the foregut mesenchyme, and spread in a craniocaudal direction throughout the gastrointestinal tract 21. In humans, the process takes about 7 weeks, with neural crest derivatives entering the foregut at 5 weeks, reaching the distal ileum by 7 weeks, the midcolon by 8 weeks, and taking a further 4 weeks to reach the distal rectum 22. It is the longest journey for cells reaching the distal rectum during embryogenesis. Therefore, it is not surprising that any factor that affects proliferation, survival, migration, or differentiation of ENCCs might result in aganglionosis.

Recently, a number of genes have been found to regulate ENCC proliferation, migration and differentiation in HSCR including *RET*, *ASCL1* and *HOXB5* 23,24. Consequently, the rostrocaudal migration of ENCCs along the long axis of the embryonic and foetal bowel has been used as a model system to investigate the cellular and molecular mechanisms underlying neural crest cell migration 25. *RET*, as one of the most important disease-related genes in HSCR, has been reported to be markedly associated with cell migration. All these studies suggest that alterations in cell proliferation and migration are crucial events in the pathogenesis of HSCR. In addition, the expression level of RET has been proved highly associated with the mutation of RET. In our study, the mutation of RET in patients was also screened. The results indicated only two patients with c.352delC mutation by using Illumina HumanExome Bead Chips (data not shown). Thus, we thought that there might be other factors involved in the down-regulation of RET.

The discovery of miRNAs provides a new layer of gene regulation, which modulates complex physiological or disease phenotypes by regulating entire functional networks. In this study, we used prediction methods to find that miR-218-1 targets both *RET* and *PLAG1*. We further validated these targets experimentally. Besides, the expression of miR-218-1 was negatively correlated with *RET* and *PLAG1* expression in HSCR tissue samples, suggesting a clinical implication. To determine the functional relevance of miR-218-1, we performed cell proliferation and migration assays *in vitro*. Our results showed that miR-218-1 inhibited cell proliferation and migration *via* directly targeting *RET* and *PLAG1*. More importantly, we confirmed this pathway *in vivo*. We also detected the ganglion cell of the transgenic mice afterbirth. The defect we detected after birth kept consist with the results of the mice during embryogenesis. Unfortunately, the defect was not clearly visible in the colon of mice afterbirth, however, the ganglion cells was decreased in the miR-218-1 transgenic mice compared with the wild-type according to the results of the detailed stain of the colon. The miR-218-1 transgenic mice showed the remarkable decrease in gangliocyte as well as the compensatory hyperplasia of the nerve fibres. It has been reported that cells transfected with siRNA of *RET* show an impaired ability of migration, while knockdown of *PLAG1* in mice could cause growth retardation and reduced fertility 26. Our result is consistent with those data.

*SLIT2*/*ROBO1* signalling has been demonstrated to be angiogenic under certain circumstances and *SLIT2*/*ROBO1* interaction inhibits cell migration and promotes cell death 27. Here, we showed that SLIT2-N, the functional subunit of SLIT2 suppressed cell migration, whereas knockdown of ROBO1 reversed such inhibitory effect, suggesting *SLIT2*/*ROBO1* pathway is also involved in the pathogenesis of HSCR. Moreover, overexpression of both miR-218-1 and SLIT2-N achieved maximum migration suppression compared with, miR-218-1 or *SLIT2* alone; suggesting the progress of the disease is regulated by multiple pathways.

**Figure 7:**
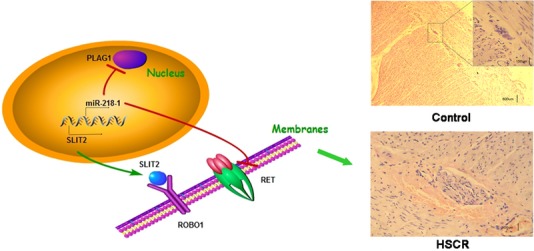
Proposed mechanism underlying pathogenesis of HSCR. Aberrant expression of SLIT2 inhibits cell migration *via*ROBO1 as well as miR-218-1-RET/PLAG1 pathway, resulting in aganglionosis which corporately contributes to the development of HSCR disease.

MiR-218-1, acting as an important factor involved in the pathogenesis of HSCR, might cause the decreased level of RET induced the loss of the ganglion cells resulting in HSCR. Besides, the down-regulation of PLAG1 was also associated with the aberrant up-regulation of miR-218-1 which was related with the loss of ganglion cells. In addition, SLIT2, the host gene of miR-218-1, could also regulate the receptor ROBO1, reducing the migration of ganglion cells. Taken together, this study reveals a novel mechanism in the pathogenesis of HSCR (Fig.7). Aberrant expression of SLIT2 inhibits cell migration *via* ROBO1 as well as miR-218-1-RET/PLAG1 pathway, which corporately contributes to the development of HSCR disease. The new factors involved in this pathway might be new targets for the early diagnosis or target therapy of HSCR.

## Supporting Information

**Figure S1** The efficiency detection of transfection assay.

**Figure S2** Results with no statistics difference in cells treated with recombinant SLIT2-N.

**Figure S3** Decreased level of PLAG1 induced a suppression of proliferation *in vitro*.

**Figure S4** Integrated density analysis of the bands in Western blot assays.

**Figure S5** Generation of miR-218-1 transgenic mice and the detection of miR-218-1 levels.

**Table S1** The target genes of miR-218-1 after predicting with bioinformatics software. (Predicted by miRanda, PicTar, TargetScan and miRNA Target database respectively).

**Table S2** Sequences of primers for RT-PCR and miR-218, siRNA-related sequence.
