# SLIT2/ROBO1‐miR‐218‐1‐RET/PLAG1: A new disease pathway involved in Hirschsprung's disease

**DOI:** 10.1111/jcmm.15092

**Published:** 2020-05-07

In Tang et al,^1^ SLIT2/ROBO1‐miR‐218‐1‐RET/PLAG1: a new disease pathway involved in Hirschsprung's disease. J. Cell. Mol. Med., 19:1197‐1207. 10.1111/jcmm.12454, the original article contains incorrect Figure 5B. The correct version of Figure 5 (panel B) should have been as depicted below. The authors confirm all results and conclusions of this article remain unchanged.

**FIGURE 5:**
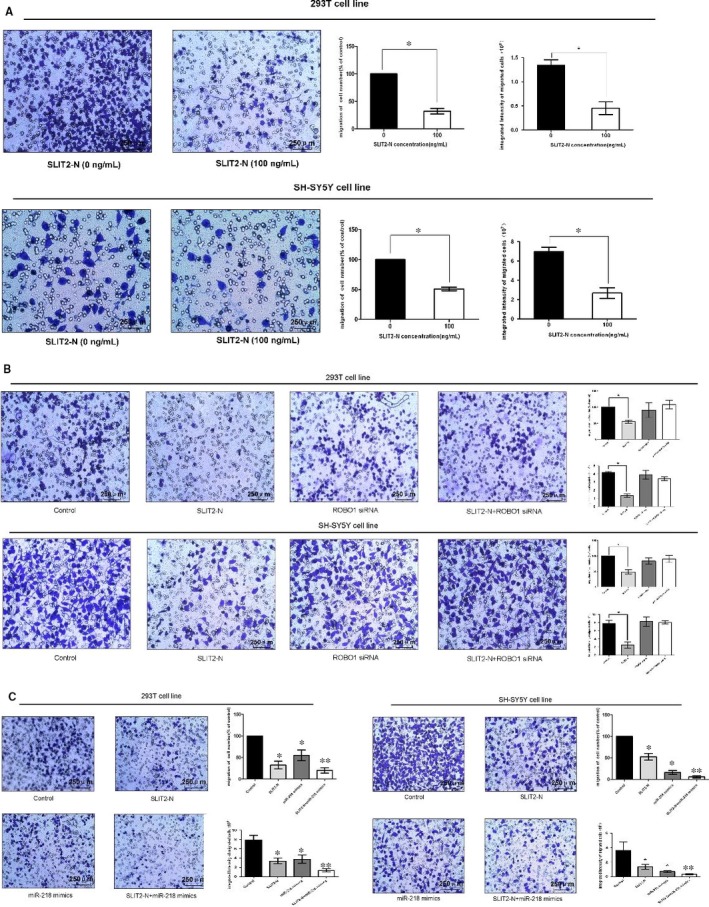
Overexpression of SLIT2 *via* SLIT2/ROBO1 pathway combining with miR‐218‐1 suppressed cell migration. (A) Transwell assay was performed as described in Materials and methods. The representative images of invasive cells at the bottom of the membrane stained with crystal violet were visualized as shown (left). The quantifications of cell migration were presented as percentage migrated cell numbers and the integrated intensity of migrated cells (right). (B) Cells were treated with 100 ng/ml SLIT2‐N, ROBO1 siRNA, SLIT2‐N + ROBO1 siRNA and normal control for 48 hrs. The representative images of invasive cells at the bottom of the membrane stained with crystal violet were visualized as shown (left). The quantifications of cell migration were presented as the percentage of migrated cell numbers and the integrated intensity of migrated cells (right). (C) Cells were treated with 100 ng/ml SLIT2‐N, miR‐218 mimics, SLIT2‐N + miR‐218 mimics and normal control for 48 hrs. The representative images of invasive cells at the bottom of the membrane stained with crystal violet were visualized as shown (left). The quantifications of cell migration were presented as percentage migrated cell numbers and the integrated intensity of migrated cells (right). All experiments were performed in triplicate and presented as mean ± SE. *indicates significant difference compared with control group (*P* < .05)

The authors wished to apologize for any misunderstanding or inconvenience this may have caused.
