# Correction to “SLIT2/ROBO1‐miR‐218‐1‐RET/PLAG1: A New Disease Pathway Involved in Hirschsprung's Disease”

**DOI:** 10.1111/jcmm.70884

**Published:** 2025-10-17

Tang W, Tang J, He J, et al. SLIT2/ROBO1‐miR‐218‐1‐RET/PLAG1: a new disease pathway involved in Hirschsprung's disease. *Journal of Cellular and Molecular Medicine* 19, no. 6 (2015): 1197–1207, https://doi.org/10.1111/jcmm.12454.

In Tang et al., the schematic diagram of the Transwell assay in Figure 3A had an inadvertent typesetting oversight during the preparation of the figure. This correction does not affect the conclusions drawn in the study.

The correct Figure 3 is shown below.

**FIGURE 3:**
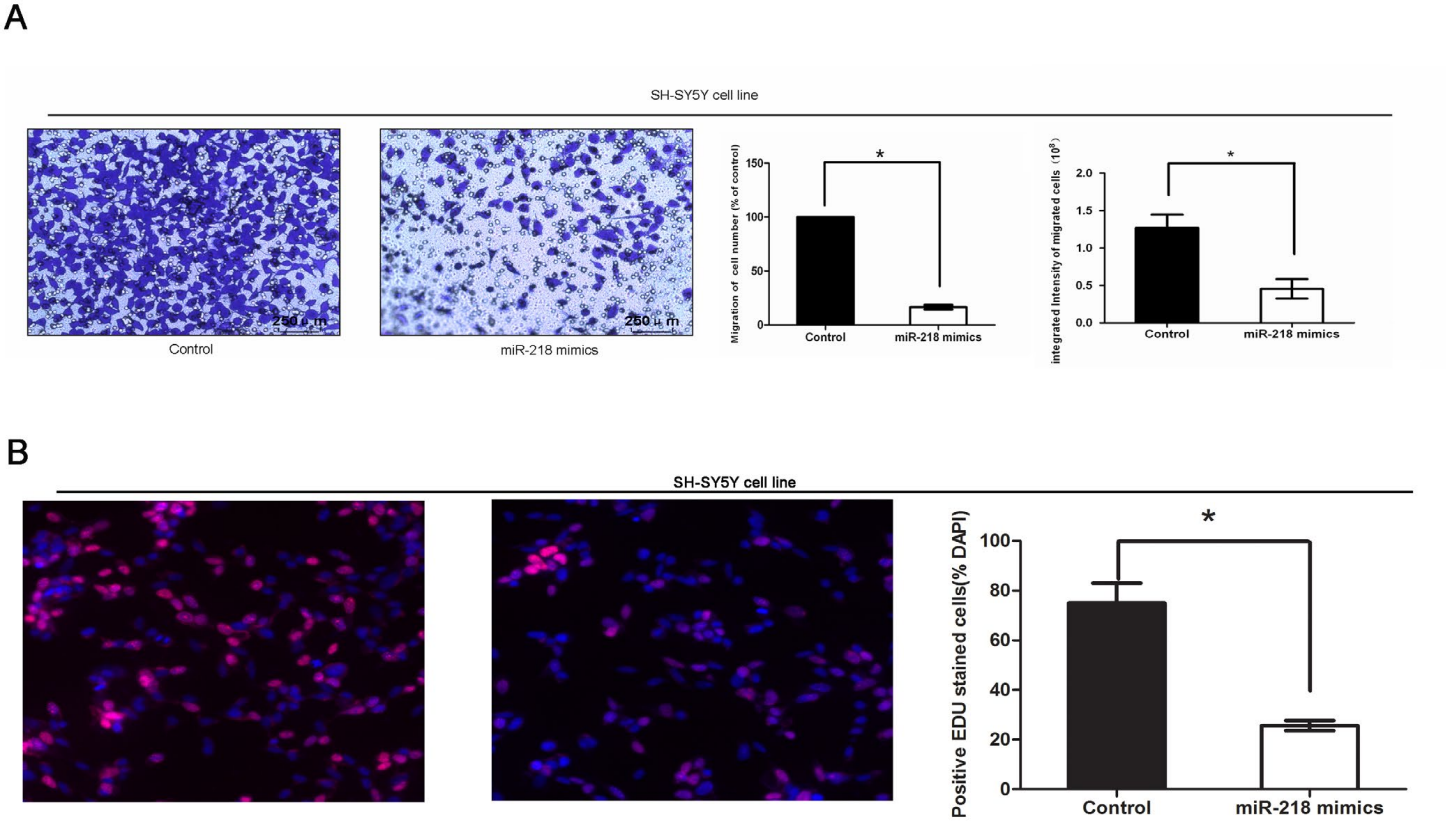
Cytobiology changes after treating cells with *miR‐218* mimics. (A) Transwell assay was performed as described in the ‘Materials and methods’ section. The representative images of invasive cells at the bottom of the membrane stained with crystal violet were visualized as shown (left). The quantifications of cell migration were presented as percentage migrated cell numbers and the integrated intensity of migrated cells (right). * indicates a significant difference compared with the control group (*p* < 0.05). (B) EDU assay was performed as described. The integrated density was presented with mean SE. * indicates a significant difference compared with the control group (*p* < 0.05).

