# Supplementary figures and images for: SLIT2/ROBO1-miR-218-1-RET/PLAG1: a new disease pathway involved in Hirschsprung's disease
Source: J Cell Mol Med. 2015 Mar 19;19(6):1197–207. doi: 10.1111/jcmm.12454 (PMC4459835; doi:10.1111/jcmm.12454)

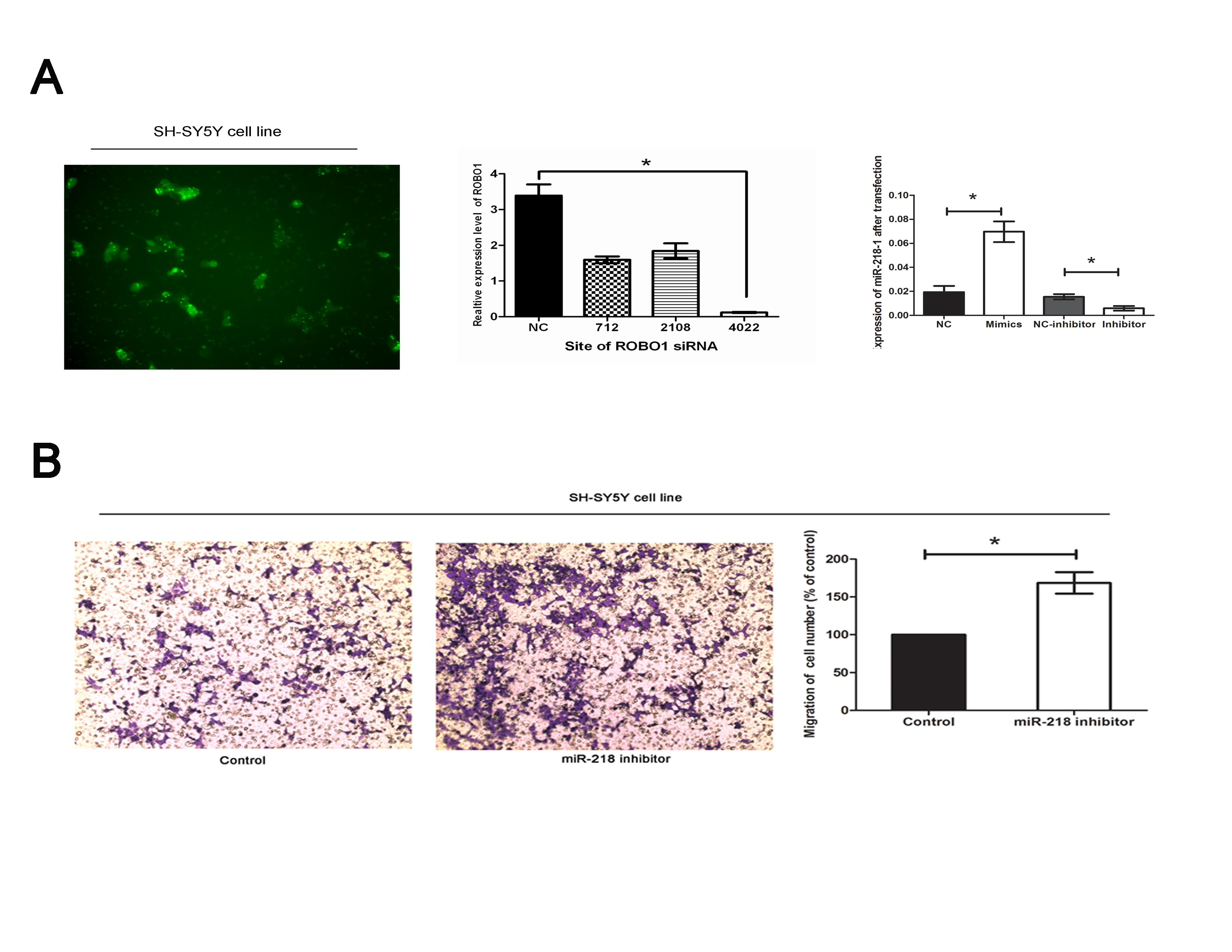

Supplement: Supplementary file 1 [file jcmm0019-1197-sd1.tif]

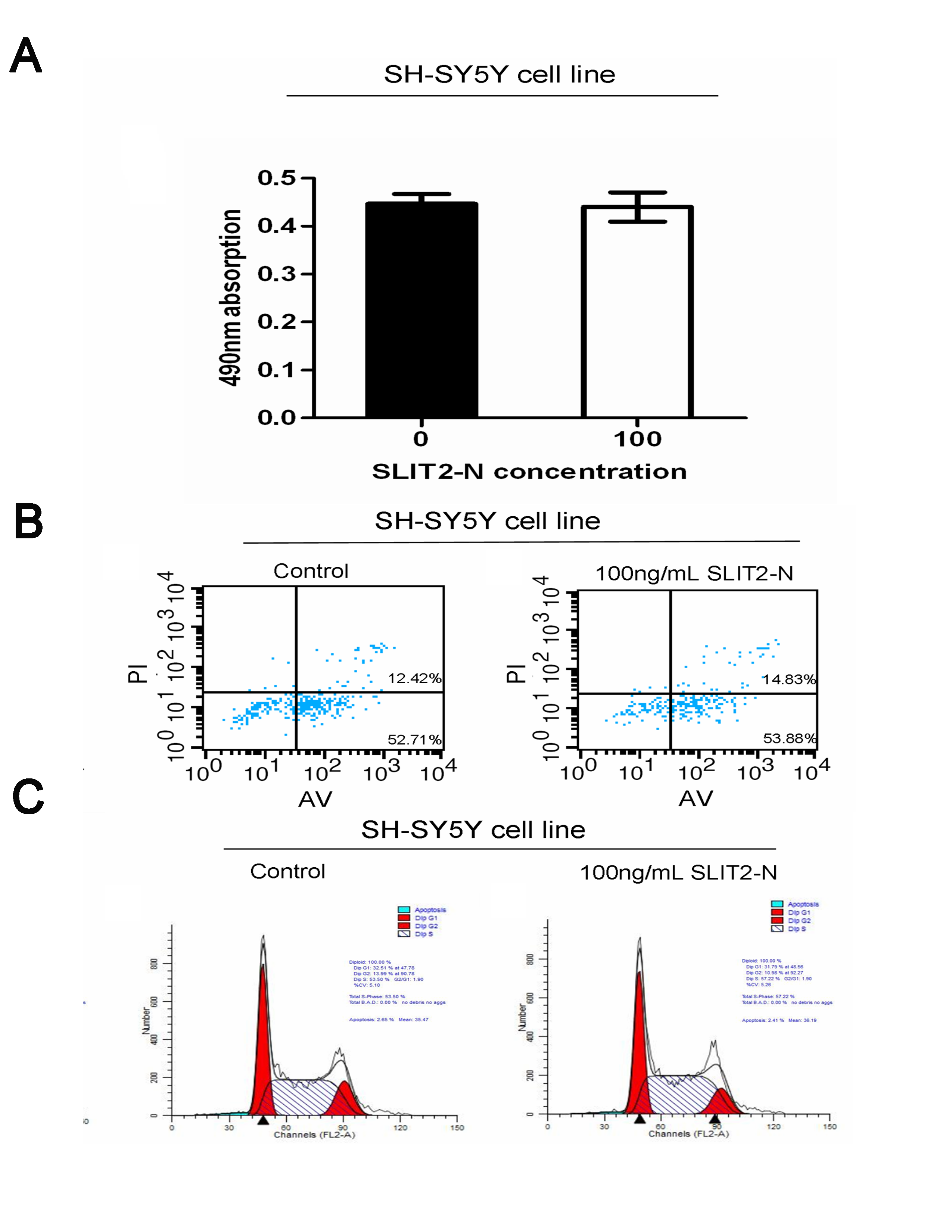

Supplement: Supplementary file 2 [file jcmm0019-1197-sd2.tif]

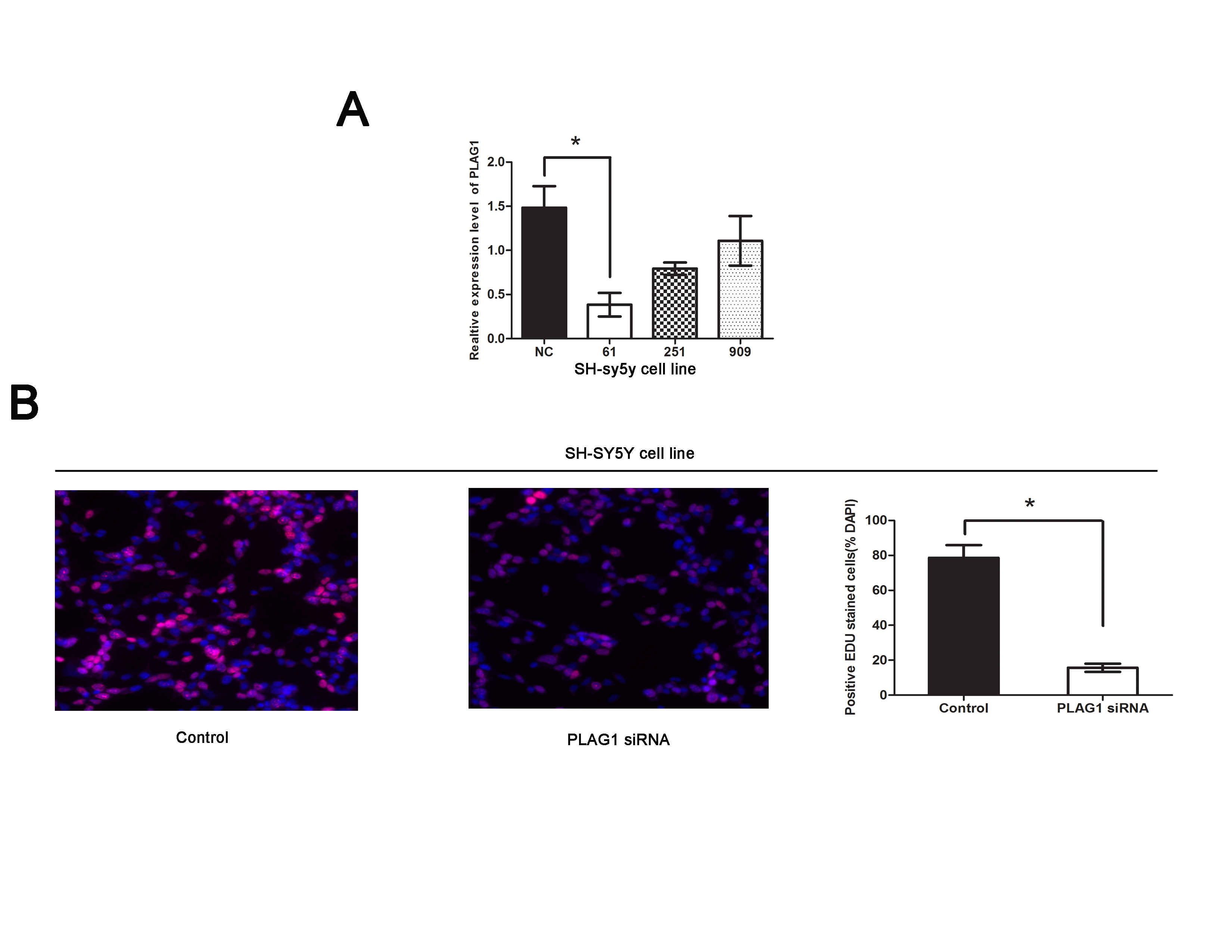

Supplement: Supplementary file 3 [file jcmm0019-1197-sd3.tif]

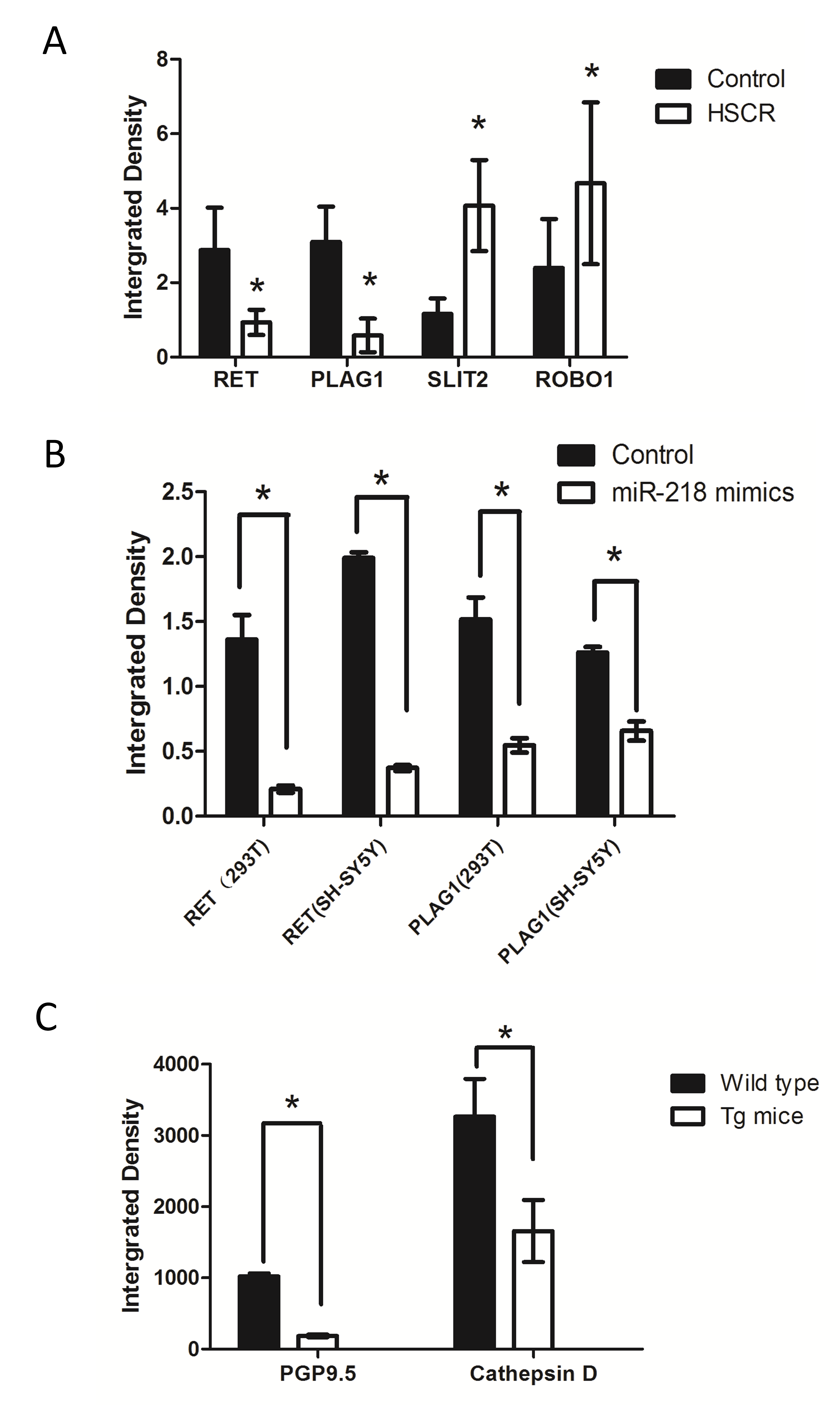

Supplement: Supplementary file 4 [file jcmm0019-1197-sd4.tif]

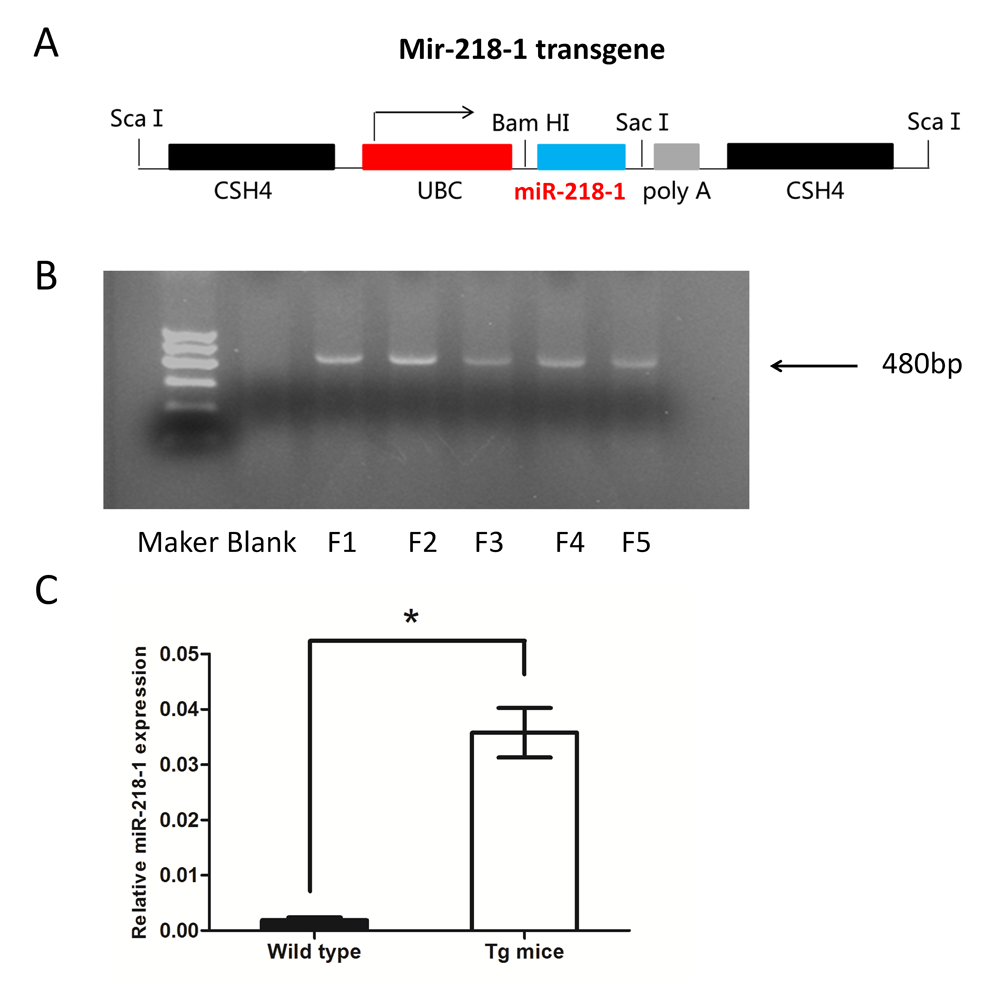

Supplement: Supplementary file 5 [file jcmm0019-1197-sd5.tif]
