# Supplementary material for: SLIT2/ROBO1-miR-218-1-RET/PLAG1: a new disease pathway involved in Hirschsprung's disease
Source: J Cell Mol Med. 2015 Mar 19;19(6):1197–207. doi: 10.1111/jcmm.12454 (PMC4459835; doi:10.1111/jcmm.12454)
Supplement: Supplementary file 6 [file jcmm0019-1197-sd6.doc]

**Table S1** Bioinformatics: Predict potential targets of miR-218-1.

| microRNA | Related Gene | Target Scan | PicTar | miRNA.org |
| --- | --- | --- | --- | --- |
| hsa-miR-218-1 | RET | **√** | **√** | **√** |
|  | SLC1A2 | **√** | **√** | **√** |
|  | TMEM25 | **√** | **√** | **√** |
|  | ZDHHC8 | **√** | **√** | **√** |
|  | EIF5A2 | **√** | **√** |  |
|  | PLAG1 | **√** | **√** |  |
