# Supplementary material for: SLIT2/ROBO1-miR-218-1-RET/PLAG1: a new disease pathway involved in Hirschsprung's disease
Source: J Cell Mol Med. 2015 Mar 19;19(6):1197–207. doi: 10.1111/jcmm.12454 (PMC4459835; doi:10.1111/jcmm.12454)
Supplement: Supplementary file 7 [file jcmm0019-1197-sd7.doc]

**Table S2** Sequences of primers for qRT-PCR and miR-218, siRNA related sequence.

| **Primers used for mRNA detection** |
| --- |
| *RET* Forward 5'-caccacgcaaagtgatgtatg-3' |
| Reverse 5'-ccctagggtcacgatctcc-3' |
| *PLAG1*  Forward 5'-ATCACCTCCATACACACGACC-3' |
| Reverse 5'-AGCTTGGTATTGTAGTTCTTGCC-3' |
| *SLIT2*   Forward 5'-CACCTCGTACAGCCGCACTT-3' |
| Reverse 5'-TGTGGACCGCTGAGGAGCAA-3' |
| *ROBO1*  Forward 5'-CGCCCCACACCCACTATTG-3' |
| Reverse 5'-GAAGTCATCCCGAAGTATGGC-3' |
| *β-actin* Forward 5'-ccaaccgcgagaagatga-3' |
| Reverse 5'-ccagaggcgtacagggatag -3*'* |
| **miRNA sequences** |
| miR-218 mimics Forward 5'-UUGUGCUUGAUCUAACCAUGU-3*'* |
| Reverse 5'-AUGGUUAGAUCAAGCACAAUU-'3 |
| Negative control Forward 5'-UUCUCCGAACGUGUCACGUTT-3*'* |
| Reverse 5'-ACGUGACACGUUCGGAGAATT-3*'* |
| *ROBO1* siRNA(4022) Forward 5'- GCCCAUGUUACAGGAUUGUTT-3*'* |
| Reverse 5'- ACAAUCCUGUAACAUGGGCTT-3*'* |
