# Supplementary material for: SLIT2/ROBO1-miR-218-1-RET/PLAG1: a new disease pathway involved in Hirschsprung's disease
Source: J Cell Mol Med. 2015 Mar 19;19(6):1197–207. doi: 10.1111/jcmm.12454 (PMC4459835; doi:10.1111/jcmm.12454)
Supplement: Supplementary file 8 [file jcmm0019-1197-sd8.doc]

**Supplement materials**

**Bioinformatics analysis**

The method we used to predicted the related potentially target RET was according to the prediction of the bioinformatical software online including Target Scan (www.targetscan.org), PicTar (pictar.mdc-berlin.de/) and miRNA.org (http://www.microrna.org/microrna/getMirnaForm.do)

**Transgenic mice**

A fragment of DNA containing the precursor sequence of mmu-miR-218-1 was amplified and subcloned into the ScaI and BamHI sites of the pUBC CSH4 mMir218-1 constructs carrying the UBC promoter and BGH poly(A) signal. The schematic diagram was presented below. Transgenic mice were generated by pronuclear injection of the transgene into the C57BL/6 strain. Genomic DNA isolated from the tail was analyzed. The positive miR-218-1 transgenic mice were identified by the successful PCR amplification. The detailed primer was MIR218-EF3/BGH-R (MIR218-EF3: 5’-CGAGGCAGGTCTTACTTGTCT-3’ BGH-R: 5’-AGAAGGCACAGTCGAGG-3’) with a product of 480bp under the PCR condition of 94°C for 5 min, then 35 cycles of 94°C for 30 sec, 55°C for 30sec,and 72°C for 30sec; 72°C, 10min. The number of mice in F1 was total 83. 15 out of 83 presented the successful over-expression of miR-218-1. Only one presented the postnatal death.

**Additional Figure legend**

**Figure S1. The efficiency detection of transfection assay.**

(A): The transfection efficiency was detected with fluorescence photograph to guarantee at least 50% cells were tranfected efficiently. (B): Three different siRNA oligonucleotides for ROBO1 inhibition were designed, after detecting the mRNA expression level of ROBO1; we chose 4022 site as the most efficient one. (C): Expression level of miR-218-1 in cells treated with mimics, inhibitor and controls. (D): The migration of cells was measured by Transwell assay. Cells treated with miR-218 inhibitor indicated a less migrated cells comparing with control group. * indicates significant difference compared with that of control cells (P<0.05). All tests were performed in triplicate and presented as mean ± SE.

**Figure S2. Results with no statistics difference in cells treated with recombinant SLIT2-N.**

A: No obvious different was obtained in cell proliferation in MTT assay in SH-SY5Y cell line. B: Recombinant SLIT2-N can not influence either cell apoptosis or cell cycle in SH-SY5Y cell lines.

**Figure S3. Decreased level of PLAG1 induced a suppression of proliferation in vitro**

(A) : PLAG1 was knocked-down by siRNA in SH-SY5Y cell line by Three different siRNA oligonucleotides. (B): EDU assay was performed to detect the cell proliferation; the ratio of proliferative phase cell was less in cells treated with PLAG1 siRNA comparing with the control group. * indicates significant difference compared with that of control cells (P<0.05). All tests were performed in triplicate and presented as mean ± SE.

**Figure S4. Intergraded density analysis of the bands in western blot assays**

After analyzing with Image J software, the intergraded density of the bans in western blot assays was presented below. (A): The protein expression levels of the genes in the tissues of HSCR patients and controls. (B): The protein expression levels of RET and PLAG1 in miR-218-1 over-expressed cell lines. (C): The intergrated density of PGP9.5 and Cathepsin D in wile type and transgenic mice. * indicates significant difference compared with that of control cells (P<0.05). All tests were performed in triplicate and presented as mean ± SE.

**Figure S5.** **Generation of miR-218-1 transgenic mice and the detection of miR-218-1 levels.**

(A): Schematic illustration of generation of miR-218-1 transgenic mice. (B): Successful PCR amplification level of miR-218-1 in miR-1 transgenic mice (C): The RT-PCR was performed to detect the expression levels of miR-218-1 in hindgut tissues. * indicates significant difference compared with that of control cells (P<0.05). All tests were performed in triplicate and presented as mean ± SE.

**Table S1** **The target genes of miR-218-1 after predicting with bioinformatics software. (Predicted by miRanda, PicTar, TargetScan and miRNA Target database, respectively).**

**Table S2 Sequences of primers for RT-PCR and miR-218, siRNA related sequence.**
